# Artificial shade as a heat abatement strategy to grazing beef cow-calf pairs in a subtropical climate

**DOI:** 10.1371/journal.pone.0288738

**Published:** 2023-07-19

**Authors:** Gleise M. Silva, Jimena Laporta, Federico Podversich, Tessa M. Schulmeister, Erick R. S. Santos, Jose Carlos Batista Dubeux, Angela Gonella-Diaza, Nicolas DiLorenzo

**Affiliations:** 1 Department of Agricultural, Food and Nutritional Science, University of Alberta, Edmonton, AB, Canada; 2 Department of Animal and Dairy Sciences, University of Wisconsin, Madison, WI, United States of America; 3 North Florida Research and Education Center, University of Florida, Marianna, FL, United States of America; Tokat Gaziosmanpaşa University: Tokat Gaziosmanpasa Universitesi, TURKEY

## Abstract

Grazing livestock in subtropical and tropical regions are susceptible to prolonged exposition to periods of extreme environmental conditions (i.e., temperature and humidity) that can trigger heat stress (HS). Currently, there is limited information on the effects of HS in the cow-calf sector globally, including in the southern U.S., as well as on mitigation strategies that could be implemented to improve animal well-being and performance. This study evaluated the impact of artificial shade (SHADE vs. NO SHADE) and breed (ANGUS vs. BRANGUS) on performance of pregnant-lactating cows, nursing heifers, and their subsequent offspring. Twenty-four Angus and 24 Brangus black-hided cows [579 ± 8 kg body weight (BW); approximately 85 d of gestation] and their nursing heifers (approximately 174 d of age) were randomly allocated to 12 ‘Pensacola’ bahiagrass pastures (*Paspalum notatum* Flüggé; 1.3 ha, n = 4 pairs/pasture), with or without access to artificial shade [NO SHADE BRANGUS (NSB), NO SHADE ANGUS (NSA), SHADE BRANGUS (SB), and SHADE ANGUS (SA)] for 56 d that anticipated weaning during the summer season in Florida. Body condition score (BCS) of cows, blood samples, and BW of cow-calf pairs were obtained every 14 d during the 56-d experimental period until weaning. Following weaning (d 56), treatments were ceased, and cows and weaned heifers were managed alike. Weaned heifers were randomly allocated to 4 pens (n = 12/pen) equipped with GrowSafe feed bunks for 14 d to assess stress responses during weaning via plasma haptoglobin. An effect of SHADE × BREED interaction was detected for cow ADG, BW change, final BW, and final BCS, where SB had the greatest ADG, BW change, final BW, and final BCS. On d 14, SA cows had the greatest concentrations of insulin whereas on d 28 NSB had the lowest concentrations, NSA the greatest, and SA and SB being intermediate. On d 56, SA tended to have the greatest plasma insulin concentrations and SB the lowest. Weight gain per area (kg/ha) tended to be 11.4 kg/ha greater in SHADE vs. NO SHADE pastures. Pre-weaning calf ADG tended to be 0.14 kg greater for SHADE vs. NO SHADE calves. Weaning weight and BW at 14-d post-weaning were lesser for NSB vs. NSA, SA, and SB, whereas no differences in postweaning ADG or haptoglobin were observed. Effects of SHADE × BREED × day interaction was detected on plasma concentrations of IGF-1, in which NSA heifers had the lowest concentrations on weaning day. Gestation length was greater for SHADE vs. NO SHADE cows, but with no impacts on subsequent calf birth and weaning weight. In summary, providing artificial shade to pregnant-lactating beef cows increased body weight gain of nursing heifers and Brangus cows, while no impact on Angus dams were observed. The provision of artificial shade during the first trimester of gestation did not alter growth performance of the subsequent offspring at birth and weaning even though gestation length was longer.

## Introduction

Heat stress (HS) is common during summertime in many parts of the world, including the United States [1], due to direct exposure of cattle to extreme environmental factors (i.e., increased solar radiation, humidity, and temperature). Beef cattle attempt to maintain their body temperature at approximately 38.5°C [2] by regulating internal heat production and external heat gain and heat loss [3]. However, cattle become heat stressed when their heat load or heat production is greater than their ability to dissipate heat to the environment.

It is expected that approximately 70% of the world beef supply comes from subtropical and tropical regions, including the southern United States [4]. *Bos Indicus* and *Bos Indicus x Bos Taurus* crossbred cattle, such as Brangus, are widely introduced into beef cattle herds in these areas [5], given those breeds are better adapted to subtropical and tropical conditions. In addition, *Bos-indicus* crossbred cattle utilize better low-quality forages, which are widely available in those climates, such as bahiagrass, compared with *B*. *taurus* breeds [4].

Environmentally induced hyperthermia or HS decreases efficiency of production in livestock [6]. Mitigating the effects of HS could be achieved by protecting cattle from direct solar radiation exposure with the use of natural or artificial shade [7]. Adding shade structures in pasture is the most economical means of mitigating HS in grazing animals [2]. We previously demonstrated that providing artificial shade during summer to grazing beef heifers was effective in reducing vaginal temperatures, increasing rumination and lying time, resulting in an additional 0.20 kg gain per day [8]. Our results highlighted the positive effects of implementing artificial shaded structures on pasture-based systems during summer in subtropical climates. Nevertheless, there is still a lack of information on the effectiveness and potential benefits of providing artificial shade during summer to pregnant lactating beef cows and their nursing calf and subsequent offspring.

Therefore, the implementation and development of strategies to mitigate HS in grazing livestock, such as the provision of artificial shade, and identifying its benefits on crossbred cattle are needed. We hypothesized that pregnant lactating beef cows and their nursing calves grazing bahiagrass pastures for 56-d prior to weaning during summer would benefit from the access to artificial shade by reducing core body temperature and improving body weight in cows and their calves compared to cow-calf pairs without access to shade (e.g., exposed to direct solar radiation). In addition, we hypothesized that Brangus would have greater performance compared to Angus cows as *Bos taurus* cattle are known for their impaired performance compared to *Bos indicus* crossbred cattle when consuming low-quality roughages. Specific objectives were to evaluate the effects of shade provision on vaginal temperature, blood parameters, maintenance of body weight during early gestation, and performance of the offspring of pregnant lactating beef cows, and blood parameters and growth performance of nursing calves during the pre- and post-weaning phase.

## Materials and methods

### Experimental design, animals, and treatments

The Institutional Animal Care and Use Committee of the University of Florida (protocol #201910767) approved all procedures for the experiment conducted at the North Florida Research Education Center (NFREC; Marianna, FL).

Twenty-four Angus (588 ± 8 kg of body weight [BW] and 6.1 ± 0.4 yr of age) and 24 Brangus (564 ± 8 kg BW; 6.9 ± 0.4 yr of age) lactating pregnant (87 and 80 d average gestation length, respectively) black-hided cows and their nursing heifers (214 and 200 ± 3 kg BW and 181 and 168 ± 3 d of age for Angus and Brangus, respectively) were used in a completely randomized design with a 2 × 2 factorial arrangement of treatments [breed (ANGUS or BRANGUS) × access to shade (NO SHADE or SHADE)]. Cow-calf pairs were separated by breed, stratified by initial BW and age, and randomly allocated in 12 ‘Pensacola’ bahiagrass pastures (*Paspalum notatum* Flüggé; 1.3 ha, n = 4 pairs/pasture), with or without access to artificial shade [NO SHADE BRANGUS (NSB), NO SHADE ANGUS (NSA), SHADE BRANGUS (SB), and SHADE ANGUS (SA)]; 3 pastures/each) for 56 d during summer prior to weaning. The experiment was conducted in three phases. Phase I, which involved data collection during the cow-calf pre-weaning phase, spanned from July 2^nd^ to August 27^th^, 2019. Following this, Phase II focused on collecting calf data during the post-weaning phase that occurred between August 27^th^ and September 11^th^, 2019. Finally, Phase III involved the subsequent calf performance, starting from the first calving on December 27^th^, 2019, and continuing until the last calving on March 8^th^, 2020. No methods of sacrifice and anesthesia and/or analgesia, and efforts to alleviate suffering were needed to be applied in this study.

The shade structures were comprised of an 80% shade cloth that was 2.4 m high and measured 11 × 7.5 m in length, providing 10.3 m^2^ of shade per animal/pasture, greater than the recommendation of shade for cows and calves [9; 3.7 and 2.6 m^2^ for mature beef cows and calves, respectively]. Cows and nursing heifers were offered free choice access to water and a complete mineral and vitamin premix (BEEF FOUR PLUS, W.B. Fleming company, Tifton, Georgia; Average composition, DM basis: 22% Ca, 4% P, 18.5% NaCl, 2,500 mg/kg Zn, 1,500 mg/kg Cu, 320 mg/kg I, 27 mg/kg Se, 225,000 IU vitamin A, and 75,000 IU vitamin D3). After weaning, treatments were ceased, and all cows and weaned heifers were managed alike.

### Environmental measurements

Environmental conditions, including air temperature, relative humidity, solar radiation, and wind speed, were obtained from the University of Florida automated weather network (FAWN) during the experiment period and are summarized (d 0 to 14, d 14 to 28, d 28 to 42, and d 42 to 56) in Table 1. Temperature-humidity index (THI) was calculated based on the air temperature and relative humidity obtained from FAWN according to [10]:

THI=(1.8×T+32)−[(0.55−0.0055×RH)×(1.8×T−26)],whereT=airtemperature(°C)andRH=relativehumidity(%).

**Table 1:** Biweekly average, maximum, and minimum air temperature, relative humidity, solar radiation, and wind speed during the pre-weaning phase (July 2 to September 27, 2019) and 14-d post-weaning. Lactating pregnant beef cows and their nursing calves had access to artificial shade (11×7.3 m in length and 3 m high) or not for 56-d during the pre-weaning phase.

	Day of the experiment					
*Item*	0–14	14–28	28–42	42–56	0–56	56–70
Air temperature (°C)	27.3	26.8	28.0	27.0	27.3	27.6
Max	34.8	23.1	35.1	33.9	34.4	34.7
Min	23.2	21.6	22.9	23.1	22.8	21.5
Relative humidity (%)	84.5	80.3	80	86.3	82.9	76
Max	92	92	88	94	94	94
Min	73	67	66	77	66	69
Solar radiation (w/m^2^)	207	222	231	179	210	216
Max	260	278	270	249	283	255
Min	157	121	183	109	109	170
Wind speed (km/h)	6.6	5.1	5.9	5.9	6.0	6.3
Max	10.7	6.7	8.8	8.6	10.7	27.3
Min	3.8	3.8	4.2	4.6	3.8	0.12
THI^4^	79	78	80	79	79	78.7

^1^Data from Florida automated weather network.

^2^THI = (1.8 × T + 32) − [(0.55 − 0.0055 × RH) × (1.8 × T − 26)], where T = air temperature (°C) and RH = relative humidity (%) as described by [10].

### Cow-calf pre-weaning phase data collection (Phase I)

Initial BW of cow-calf pairs was calculated as the average of full BW on d -1 and 0, while final BW was the average of d 55 and 56 (weaning weight). Additional BW measurements were obtained every 14 d, which corresponds to d 14, 28, and 42. Cow body condition score (BCS) was assessed on d 0 and 56 of the experiment. Individual BW was obtained in the morning using a calibrated scale (Tru-Test Datamars XR5000, Canada). On d 0, days of pregnancy in cows were obtained using a portable veterinary ultrasound (MyLab™DeltaVET 250179# Esaote, Genova, Italy). The average days of gestation was estimated at 85. Cow vaginal temperature was automatically recorded every 30 min for 14 consecutive days (d 14 to 28) using temperature probes (i-button DS1921H-F5#; accuracy ± 0.065°C; Maxim, Irving, TX) placed intravaginally with a blank (hormone-free) controlled internal drug release device (Pfizer Animal Health, New York, NY). For vaginal temperature measurements, 1 cow/pasture was randomly selected to receive the temperature probes.

Blood samples from cows and calves (approximately 10 mL) were collected via jugular venipuncture into sodium-heparin (158 USP) containing tubes (Vacutainer, Becton Dickinson, Franklin Lakes, NJ) to harvest plasma on d 0, 14, 28, 42, and 56. Plasma concentrations of insulin, glucose, and prolactin, were determined for cows, whereas glucose and insulin-like growth factor 1 (IGF-1) were determined in the plasma of heifers. Additionally, blood samples were collected from heifers on d 1, 3, and 7 post-weaning to assess concentrations of haptoglobin (Hp). Plasma Hp concentrations were used as an indicator of post-weaning inflammatory response [11]. Upon collection, blood samples were immediately placed on ice and centrifuged for 15 min at 4,000 × g at 4°C. After centrifugation, plasma was transferred into polypropylene vials (12 × 75 mm; Fisherbrand; Thermo Fisher Scientific Inc., Waltham, MA), and stored at −20°C for further analyses. Plasma concentrations of Hp were determined in duplicate samples using a biochemical assay assessing haptoglobin-hemoglobin complex by the estimation of differences in peroxidase activity [12]. Inter- and intra-assay coefficients of variation of Hp were 1.9% and 1.4%, respectively. Glucose (G7521 Pointe Scientific Inc., Canton, MI; inter- and intra-assay coefficients of variation were 6.2 and 4.6%, respectively), insulin (Mercodia Bovine Insulin ELISA, Mercodia Inc., Uppsala, Sweden; inter- and intra-assay coefficients of variation were 2.6, and 6.1%, respectively), prolactin (SEA846Bo, Cloud-Clone Corp., Katy, TX; inter- and intra-assay coefficients of variation were 15.8 and 7.7%, respectively), and IGF-1 (SG100 R&D Systems, Inc., Minneapolis, MN, respectively; inter- and intra-assay coefficients of variation were 2.4 and 3.1%, respectively) were determined using available commercial kits.

### Pasture data collection and laboratory analyses (Phase I)

Every 14 d (d -4, 10, 24, and 38; evaluation 1, 2, 3, and 4, respectively), forage samples from each pasture were collected to determine forage chemical composition and canopy characteristics. Herbage mass (HM; kg DM/ha) and herbage allowance (HA; kg DM/kg BW) were calculated using the double sampling technique. The technique consists of regressing forage mass clipped to soil level from a 0.25-m^2^ quadrat (direct measurement) on aluminum disk settling heights (indirect measurement). Thirty disk settling heights were taken every 14 d in each pasture on d -4, 10, 24, and 38 of the experiment, whereas clipped samples were collected every 28 d (d -6, 22, and 50 of the experiment). Using the 3 clipped samples per pasture plus the 30 heights per pasture, different regression equations were generated throughout phase I of the experiment and used to estimate the total HM and calculate HA in each pasture. Therefore, with heights being taken every 14 d, HA was adjusted with the same frequency. The R^2^ of generated equations ranged from 0.60 to 0.66. The put-and-take method was used to maintain similar HA among treatments [13]. If the difference in HA among pastures were greater than 0.4 kg DM/kg BW, cannulated steers were used to graze the pasture with high HA for 14 d until the next calculation of HA was performed. We wanted to add heavier animals to be able to correct the HA using only one animal to avoid competition for shade if they were added to a pasture with access to shade. Adjustment of stocking rate is critical to avoid confounding effect of contrasting HA among treatments, affecting animal performance as a result. When HA is similar, animal performance should be similar, unless other factors (e.g., shade structure in the case of this study) are affecting the performance. Throughout the study, the maximum number of put-and-take needed to adjust HA was 1 per pasture. Gain per area (GPA; kg/ha) was calculated by multiplying ADG of cows and calves by stocking rate and time interval (14 d). Stocking rate was calculated by dividing animal unit per area. Then, animal unit was calculated using the total body weight per pasture, including cow-calf pairs and put-and-take, divided by 450 kg.

Forage samples collected for nutritive value were dried at 55°C in an air-circulated dryer and ground using a Wiley Mill (Model 4, Thomas-Wiley Laboratory Mill, Thomas Scientific) to pass a 2-mm stainless-steel screen. In vitro organic matter digestibility (IVOMD) was determined using the two-stage technique described by [14]. For determination of the fibrous components [neutral detergent fiber (NDF) and acid detergent fiber (ADF)], forage samples were weighed into F57 bags (Ankom Technology Corp., Macedon, NY] and analyzed for NDF, using heat-stable α-amylase and sodium sulfite, and subsequently for ADF as described by [15] in an Ankom 200 Fiber Analyzer (Ankom Technology Corp). Concentration of crude protein (CP) in the samples was determined by rapid combustion using a micro elemental N analyzer (Vario Max CN, Elementar Americas Inc., Mt. Laurel, NJ), following official method 992.15 [16].

### Calf post-weaning phase data collections (Phase II)

Following weaning (from d 56 to 70), weaned heifers were moved from pastures to the Feed Efficiency Facility (FEF) at the NFREC for a period of 14-d. Heifers were treated alike as single group and randomly placed in 4 concrete-floored pens (108 m^2^;12 heifers/pen) equipped with 2 GrowSafe feed bunks (GrowSafe System, Ltd., Airdrie, Alberta, Canada) each. Heifers were offered free choice total mixed ration (TMR) that consisted of (DM basis) 80% of commercial commodity blend (processed grain by-products, roughage products, propionic acid, butyric acid, and cotton gin by-product; AFG Feed, LLC, Donalsonville, GA) and 20% of a commercial post-weaning pellet (Purina Animal Nutrition, LLC, Arden Hills, MN). Feed samples were collected and dried in a forced-air oven for 72 h at 55°C, ground in a Wiley mill (Arthur H. Thomas Co., Philadelphia, PA) to pass a 2-mm sieve, and analyzed for nutritional composition by a commercial laboratory (Dairy One Forage Laboratory, Ithaca, NY). The TMR contained (DM basis): 82% DM, 17.2% CP, 52.5% aNDF, 40.3% ADF, 17.3% lignin, 18.2% non-fibrous carbohydrates, 5.2% crude fat, 6.9% ash, 0.57% Ca, 0.58% P, 0.25 Mg, 1.21% K, 0.188% Na, 0.29% S, 746 ppm I, 117 ppm Zn, 33 ppm Cu, 101 ppm Mn, and 1 ppm Mo.

### Subsequent calf (Phase III)

After the treatment phase (Phase I), cows were managed in a single group until subsequent weaning. At calving, birth date, birth weight, and calf sex were recorded. Weaning weight was obtained at approximately 7.6 months of age.

### Statistical analyses

The data was analyzed as a completely randomized design with a 2 × 2 factorial arrangement of treatments using the GLIMMIX procedure of SAS (SAS Institute Inc., Cary, NC, USA, version 9.4). Pasture was the experimental unit for all analyses, and pasture within breed and treatment were included as random effect. Cow initial BW was used as a covariate for final BW and final BCS (*P* = 0.002), and calf BW was covariate-adjusted for calf age (0.05 < *P* ≤ 0.10). For performance variables (i.e., BW, ADG, BCS, overall DMI, and G:F), the model included the fixed effects of SHADE, BREED, and SHADE × BREED interactions. Forage variables, cow vaginal temperature, and plasma measurements, were analyzed as repeated measures and tested for fixed effects of SHADE, BREED, day, and all resulting interactions. The lowest Akaike Information Criterion was used to select the best covariance structures. Significance was set at *P* ≤ 0.05, and tendencies declared when 0.05 ≥ *P* ≤ 0.10.

## Results

### Environmental measures

Air temperature, relative humidity, solar radiation, wind speed, and THI from d 0 to 14, d 14 to 28, d 28 to 42, d 42 to 56, d 0 to 56 (July 2 to September 10, 2019) and d 56–70 (post-weaning phase) are summarized in Table 1. Average air temperature, relative humidity, solar radiation, wind speed, and THI throughout the study were 27.3°C, 83%, 210 w/m^2^, 6 km/h, and 79, respectively.

### Gain per area, herbage mass and allowance, and chemical composition

There was no SHADE × BREED × evaluation interaction nor effects of SHADE for any of the forage measurements (*P* ≥ 0.24; Table 2). Cow-calf pairs grazing pastures with access to shade tended to have a greater GPA (*P* = 0.09). An effect of evaluation was detected for IVOMD, NDF, ADF, CP, HM, and HA (*P* < 0.001) in which chemical composition was similar within evaluations 1 and 2. However, chemical composition gradually reduced when compared to evaluations 3 and 4. Herbage mass and HA increased from evaluation 1 to 2 but gradually lowered from 2 to 4 and did not differ among treatments throughout the experiment.

**Table 2:** Herbage mass, herbage allowance, gain per area, and chemical composition of bahiagrass pastures (1.3 ha/pasture) grazed by pregnant lactating Angus and Brangus cows and their nursing calves with or without access to artificial shade (11 × 7.3 m in length and 2.4 m high) during summer (56-d; July to September).

	BREED			SHADE				Evaluation^1^					*P*-value^2^		
*Item*	ANGUS	BRANGUS	SEM	SHADE		NO SHADE	SEM	1	2	3	4	SEM	BREED	SHADE	EVAL
HM^3^, kg DM/ha															
	6986	6955	129	6984	6957		13	7479^b^	8944^a^	6013^c^	5445^d^	158	0.87	0.88	< 0.01
HA^4^, kg DM/kg of BW															
	2.1	2.2	0.03	2.1	2.1		0.03	2.38 ^a^	2.7^b^	1.8^c^	1.6^d^	0.1	0.17	0.95	< 0.01
GPA^5^, kg/ha															
	57.3	66.7	4.29	67.7	56.3		4.3	89.5^a^	73.6^ab^	53.9^b^	30.9^c^	7.1	0.15	0.09	< 0.01
IVOMD^6^%	48.8	53.3	1.0	50.3	51.8		1.0	56.6^a^	53.7^ab^	50.4^b^	43.6^c^	1.6	0.01	0.32	< 0.01
NDF^7^	64.9	61.9	1.1	63.2	64.0		0.6	57.4^a^	62.3^a^	66.9^b^	67.6^c^	0.8	0.03	0.48	< 0.01
ADF^7^	30.3	29.7	0.3	30.3	29.5		0.3	26.1^a^	28.4^a^	32.8^b^	32.7^c^	0.4	0.27	0.15	< 0.01
CP^7^	18.1	18.7	0.4	18.1	18.6		0.4	21.4^a^	20.6^a^	17.8^b^	13.6^c^	0.5	0.35	0.45	< 0.01

^a-b^Within a row, means without a common prescript differ (*P* ≤ 0.05).

^1^Evaluation 1, 2, 3, and 4 corresponds to samples collected on d -4, 10, 24, and 38 of the experiment, respectively.

^2^Effect of BREED × SHADE × evaluation, or any two-way interaction was not detected (*P* ≥ 0.06) for any of the variables.

^3^Herbage mass estimated on d -4, 10, 24, and 38 of the experiment.

^4^Herbage allowance estimated on d -4, 10, 24, and 38 of the experiment.

^5^Gain per area in kg/ha was calculated using total cow-calf body weight gain.

^6^In vitro organic matter digestibility determined using the two-stage technique described by Moore and Mott (1974) of forage samples collected on d -4, 10, 24, and 38.

^7^Neutral detergent fiber, acid detergent fiber, and crude protein of forage samples collected on d -4, 10, 24, and 38 of the experiment. Samples of NDF and ADF were determined as described by [15], whereas CP was determined using a micro elemental N analyzer (Vario Max CN, Elementar Americas Inc., Mt. Laurel, NJ), following official method 992.15 (AOAC, 1995).

### Cow performance, vaginal temperature, and plasma measurements

An effect of BREED × SHADE interaction was detected for cow ADG, BW change, final BW, and final BCS (*P* ≤ 0.05; Table 3), where SB had the greatest ADG), BW change, final BW, and final BCS. However, SA, NSA, and NB cows did not differ among them. There were no effects of BREED × SHADE interaction or main effects of SHADE or BREED for cow age (*P* ≥ 0.98), initial BW (*P* ≥ 0.06) and initial BCS (*P* ≥ 0.36). ANGUS and BRANGUS cows were 6.1 and 6.9 ± 0.4 yr old, and weighed 588 vs. 564 ± 8 kg, respectively at the start of the experiment.

**Table 3:** BREED × SHADE interaction on body weight change, average daily gain, final body weight, and final body condition score of ANGUS and BRANGUS cows and on body weight of their nursing heifers with or without access to artificial shade (11×7.3 m in length and 2.4 m high) during summer (56-d; July to September).

*Item*	SHADE		NO SHADE				
	ANGUS	BRANGUS	ANGUS	BRANGUS	SEM		*P*-value
Cow							
Final BW^1^, kg	576^b^	599^a^	579^b^	565^b^	6.0	0.01	
BW change, kg	0.2^b^	22.5^a^	3.0^b^	-9.9^b^	4.4	0.004	
Final BCS^1^, kg	4.3^b^	5.2^a^	4.6^b^	4.7^ab^	0.16	0.05	
ADG kg	0.00^b^	0.39^a^	0.06^b^	-0.18^b^	0.081	0.004	
Nursing heifers							
BW d 28, kg	240^ab^	230^bc^	249^a^	220^c^	4.4	< 0.001	
BW d 42, kg	250^a^	243^a^	247^a^	232^b^	3.6	< 0.001	
BW d 56, kg	253^a^	247^a^	259^a^	233^b^	5.1	< 0.001	
BW d 70, kg	261^a^	258^a^	268^a^	242^b^	5.1	< 0.001	

^a-c^Within a row, means without a common prescript differ (*P* ≤ 0.05).

^1^Final BW and BSC of cows at the end of the treatment period (d 56; weaning day) were covariate adjusted for initial BW (*P* < 0.05).

**Fig 1:**
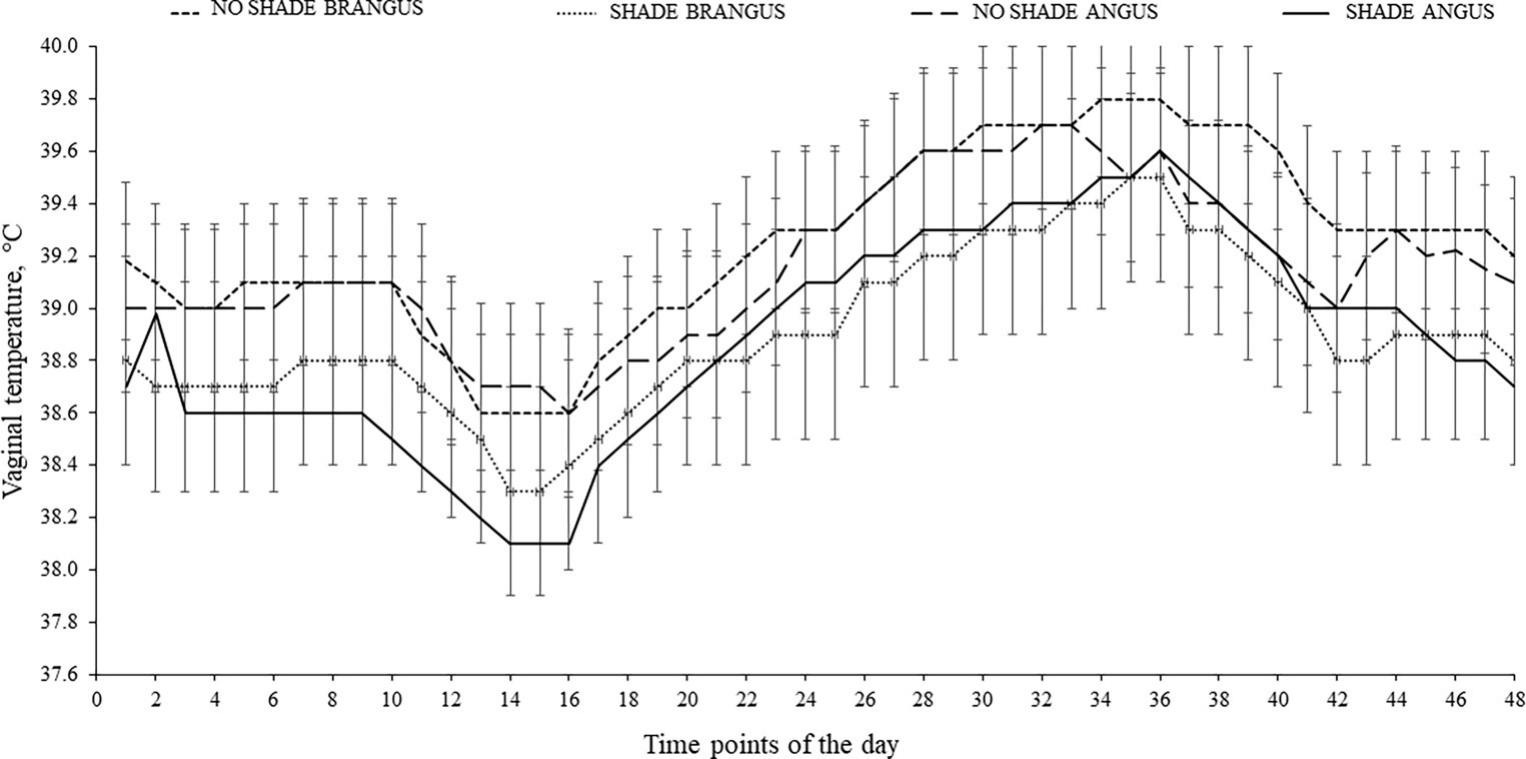
Effects of SHADE × BREED × hour interaction on vaginal temperature (*P* < 0.001; SEM = 0.39) of ANGUS and BRANGUS cows grazing bahiagrass pastures with or without access to artificial shade (11× 7.3 m in length and 2.4 m high) for 56-d during summer. Data was recorded every 30 min from d 14 to 28 (July 16–30, 2019) of the experiment. There was no difference among SHADE within hours when individual mean comparisons were evaluated (*P* > 0.11). There was, however, an effect of hour of the day on vaginal temperature (*P* < 0.001).

Effects of SHADE × BREED × hour (*P* < 0.001), BREED × hour (*P* = 0.01), and SHADE × hour (*P* = 0.006) interactions were detected on vaginal temperature of cows (Fig 1). However, such differences were not observed during the post-hoc analysis (*P* > 0.11). A significant effect of hour of the day was observed for vaginal temperature (*P* < 0.001), in which temperatures gradually increased from 0800 to 1800 h.

**Fig 2:**
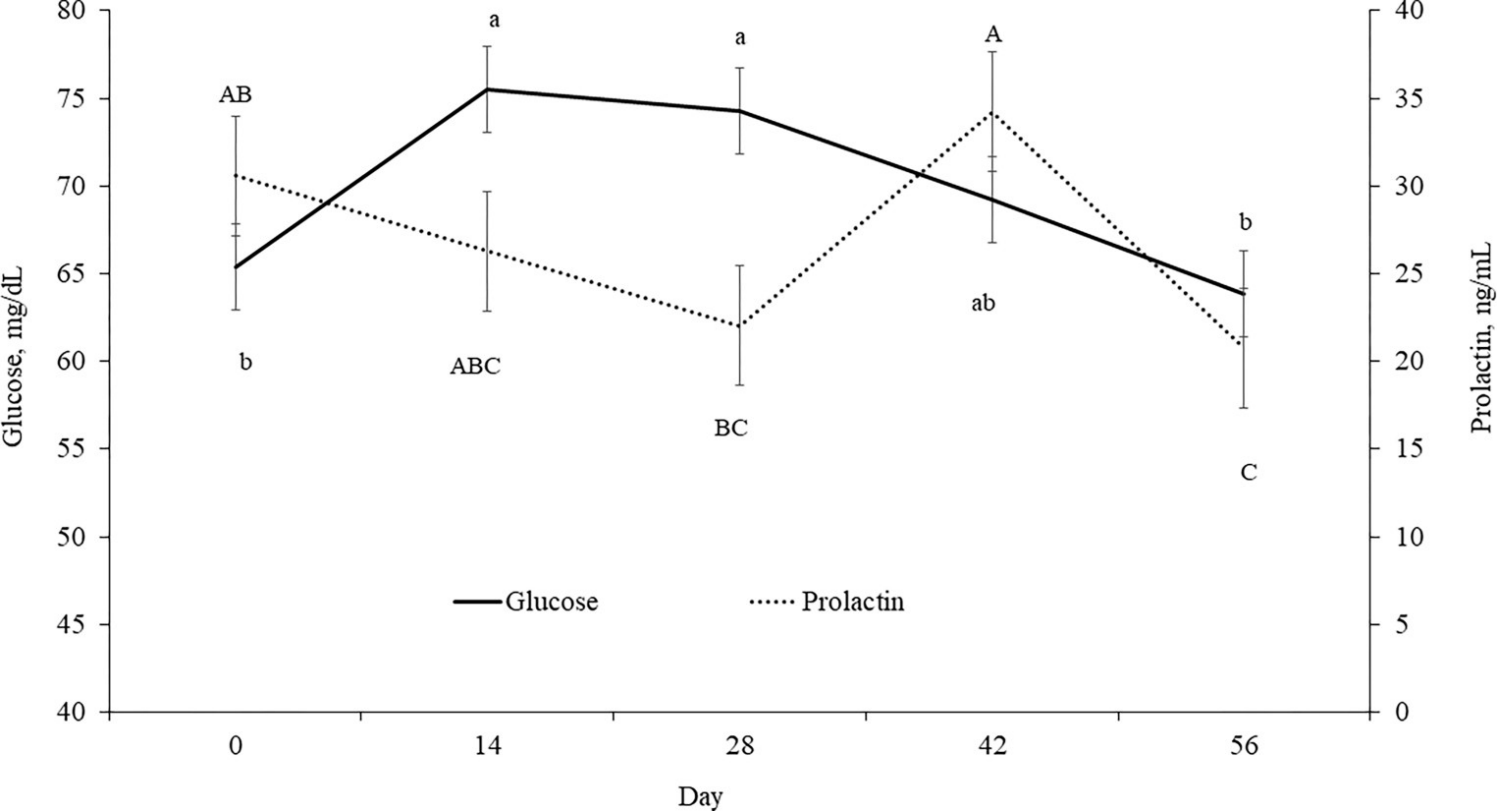
Effect of day on plasma concentrations of glucose (*P* = 0.002; SEM = 2.45) and prolactin (*P* = 0.02; SEM = 3.41) of ANGUS and BRANGUS multiparous cows grazing bahiagrass pastures with or without access to artificial shade (11× 7.3 m in length and 2.4 m high) for 56-d during summer. Within day, means without a common superscript (a–b or A-B; glucose and prolactin, respectively) differ (*P* ≤ 0.05).

No effects of SHADE were observed (*P* ≥ 0.55) for plasma concentrations of glucose and prolactin. However, plasma concentrations of glucose (*P* = 0.002) and prolactin (*P* = 0.02) in cows were affected by day (Fig 2), where glucose was greatest on d 14 and 28, whereas prolactin concentrations were the lowest on d 56. Furthermore, ANGUS cows had greater concentrations of glucose and prolactin (*P* ≤ 0.05; 72.8 vs. 66.4 ± 2.05 mg/dL and 34.0 vs. 19.4 ± 2.96 ng/mL, respectively) compared to BRANGUS cows.

**Fig 3:**
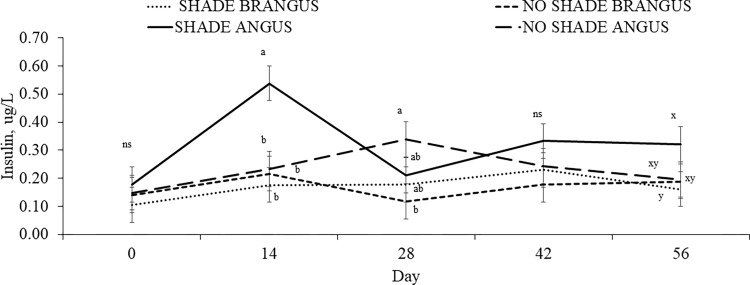
Effects of SHADE × BREED × day interaction on plasma concentrations of insulin (*P =* 0.05; SEM = 0.067) of ANGUS and BRANGUS cows grazing bahiagrass pastures with or without access to artificial shade (11× 7.3 m in length and 2.4 m high) for 56-d during summer. Means without a common superscript (a–e) differ (*P* ≤ 0.05) or tend to differ (0.05 ≥ *P* ≤ 0.10), whereas no significant differences are represented by “ns” (*P* ≥ 0.10).

Effects of SHADE × BREED × day (*P* = 0.05; SEM = 0.067) were detected for plasma concentrations of insulin (Fig 3). On d 14, SA cows had the greatest concentrations of insulin whereas on d 28 NSB had the lowest concentrations, NSA the greatest, and SA and SB being intermediate. On d 56, SA tended to have the greatest plasma insulin concentrations and SB the lowest.

### Heifer performance and plasma measurements

Heifer age and initial BW were affected by BREED (*P* ≤ 0.04; Table 4). Therefore, heifer age was used as a covariate for BW (*P* ≤ 0.05). BREED (*P* = 0.03) and provision of shade (*P* = 0.03) affected ADG from d 0 to 14, whereas the availability of shade tended to affect ADG from 0 to 56 (*P* = 0.10; Table 4).

**Table 4:** Pre-weaning growth performance of ANGUS and BRANGUS nursing heifers with or without access to artificial shade (11×7.3 m in length and 2.4 m high) during summer (56-d; July to September).

	BREED			SHADE			*P*-value		
*Item*	ANGUS	BRANGUS	SEM	SHADE	NO SHADE	SEM	BREED	SHADE	BREED × SHADE
Heifer age, d	181	168	3.45	171	178	3.47	0.04	0.18	0.26
Initial BW, kg	213	200	2.7	206	207	2.4	0.01	0.91	0.26
ADG, kg									
0 to 14	1.32	1.00	0.08	1.31	1.00	0.08	0.03	0.03	0.77
0 to 28	1.23	0.77	0.12	1.00	1.00	0.07	0.45	0.71	0.36
0 to 42	0.82	0.90	0.05	0.90	0.81	0.05	0.19	0.13	0.80
0 to 56	0.73	0.77	0.05	0.82	0.68	0.05	0.49	0.10	0.48
BW^1^, kg									
d 14	232	214	1.96	225	220	2.54	0.01	0.27	0.35
d 28	246	224	2.22	235	234	2.23	0.002	0.94	0.03
d 42	249	237	2.13	245	241	1.93	0.01	0.18	0.06
Weaning weight, kg	257	240	2.45	250	246	2.45	0.01	0.34	0.04

^1^Interim and weaning weight were covariate adjusted for calf age.

During the pre-weaning phase, heifers with access to shade gained 0.14 kg/d more than heifers without access to shade. An interaction of BREED × SHADE was detected for BW on d 28 (*P* = 0.03), 42 (*P* = 0.06), and 56 (*P* = 0.04), in which NSB heifers had the lowest BW (Tables 3 and 4). NO SHADE BRANGUS were 20, 14, and 26 ± 2.4 kg lighter on weaning day compared to SA, NSA, and SB, respectively. The differences in BW observed at weaning persisted at d 70 (14-d post-weaning; *P* < 0.001; Tables 3 and 5) even though the treatments ended on d 56.

**Table 5:** Post-weaning growth performance (d 56–70) of ANGUS and BRANGUS heifers that were exposed to pastures with or without access to artificial shade (11×7.3 m in length and 2.4 m high) for 56-d during the pre-weaning phase (July to September).

	BREED		SEM	SHADE			*P*-value		
*Item*	ANGUS	BRANGUS		SHADE	NO SHADE	SEM	BREED	SHADE	BREED × SHADE
ADG, kg	0.53	0.79	0.17	0.72	0.61	0.17	0.32	0.68	0.82
BW d 70, kg	265	250	2.91	260	255	5.64	0.02	0.30	0.03
DMI, kg/d	5.24	4.95	0.29	4.73	5.45	0.29	0.50	0.11	0.62
DMI, % BW	2.0	2.0	0.12	2.1	1.8	0.12	0.98	0.15	0.98
G:F d-56 to 70	0.13	0.21	0.04	0.21	0.13	0.04	0.15	0.18	0.17

^1^Body weight on d 70 was covariate-adjusted to calf age.

**Fig 4:**
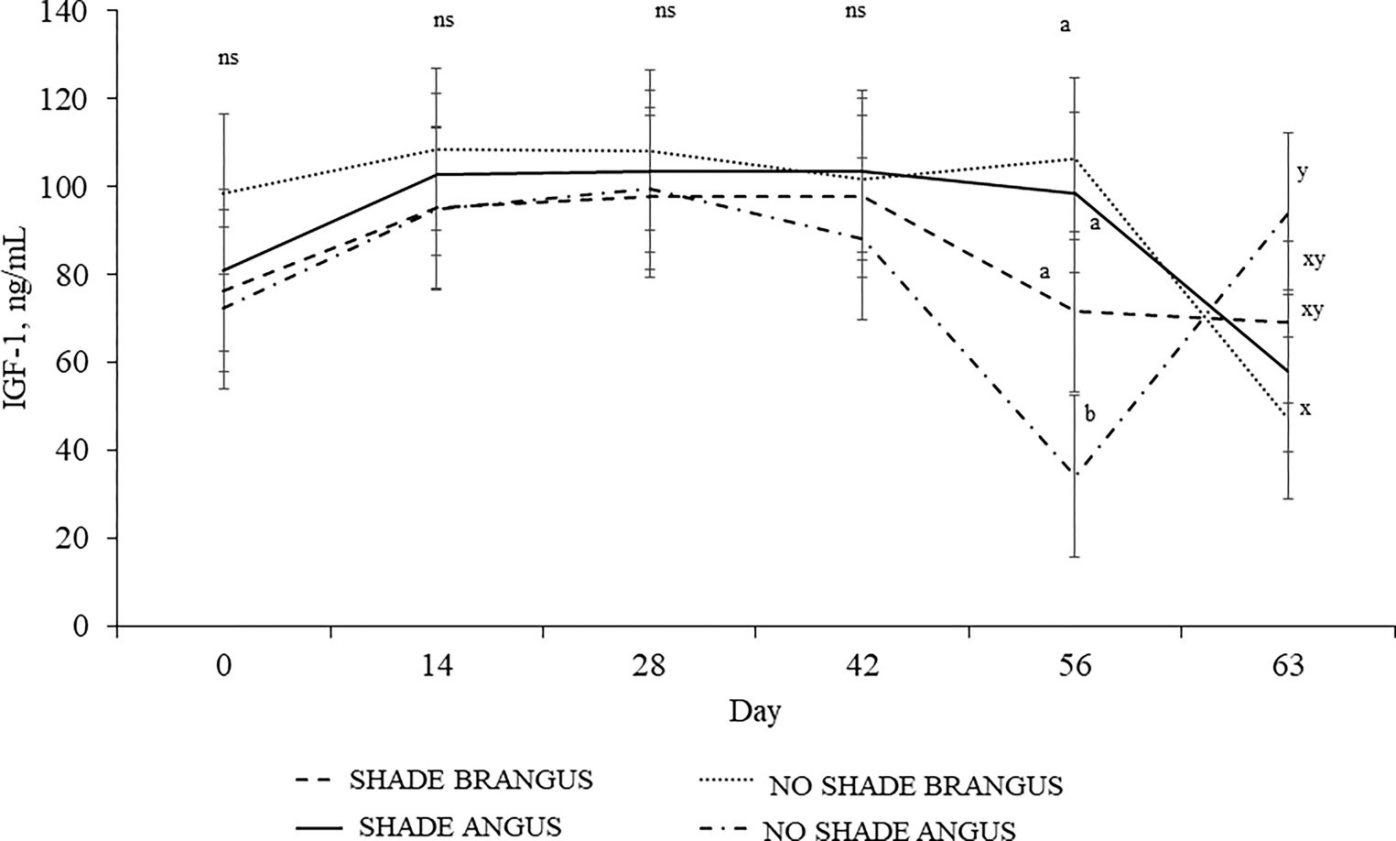
Effects of SHADE × BREED × day interaction on plasma concentrations of IGF-1 (*P =* 0.01; SEM = 18.36) of ANGUS and BRANGUS nursing heifers grazing bahiagrass pastures with or without access to artificial shade (11× 7.3 m in length and 2.4 m high) for 56-d prior to weaning. Within day, means without a common superscript (a–b) differ (*P* ≤ 0.05) or tend to differ (0.05 ≥ *P* ≤ 0.10), whereas no significant differences are represented by “ns” (*P* ≥ 0.10).

A SHADE × BREED interaction (*P* = 0.04) and tendency for an effect of day (*P* = 0.08) was observed for plasma concentrations of glucose. NO SHADE BRANGUS heifers had the greatest concentration of glucose, whereas NSA the lowest, and SA and SB were intermediate (110.6, 85.5, 92.2, 87.0 ± 6.4 mg/dL for NSB, NSA, SA, and SB, respectively). Effects of SHADE × BREED × day interaction was detected on plasma concentrations of IGF-1 (*P* = 0.01; Fig 4), in which NSA heifers had the lowest concentrations on weaning day.

**Fig 5:**
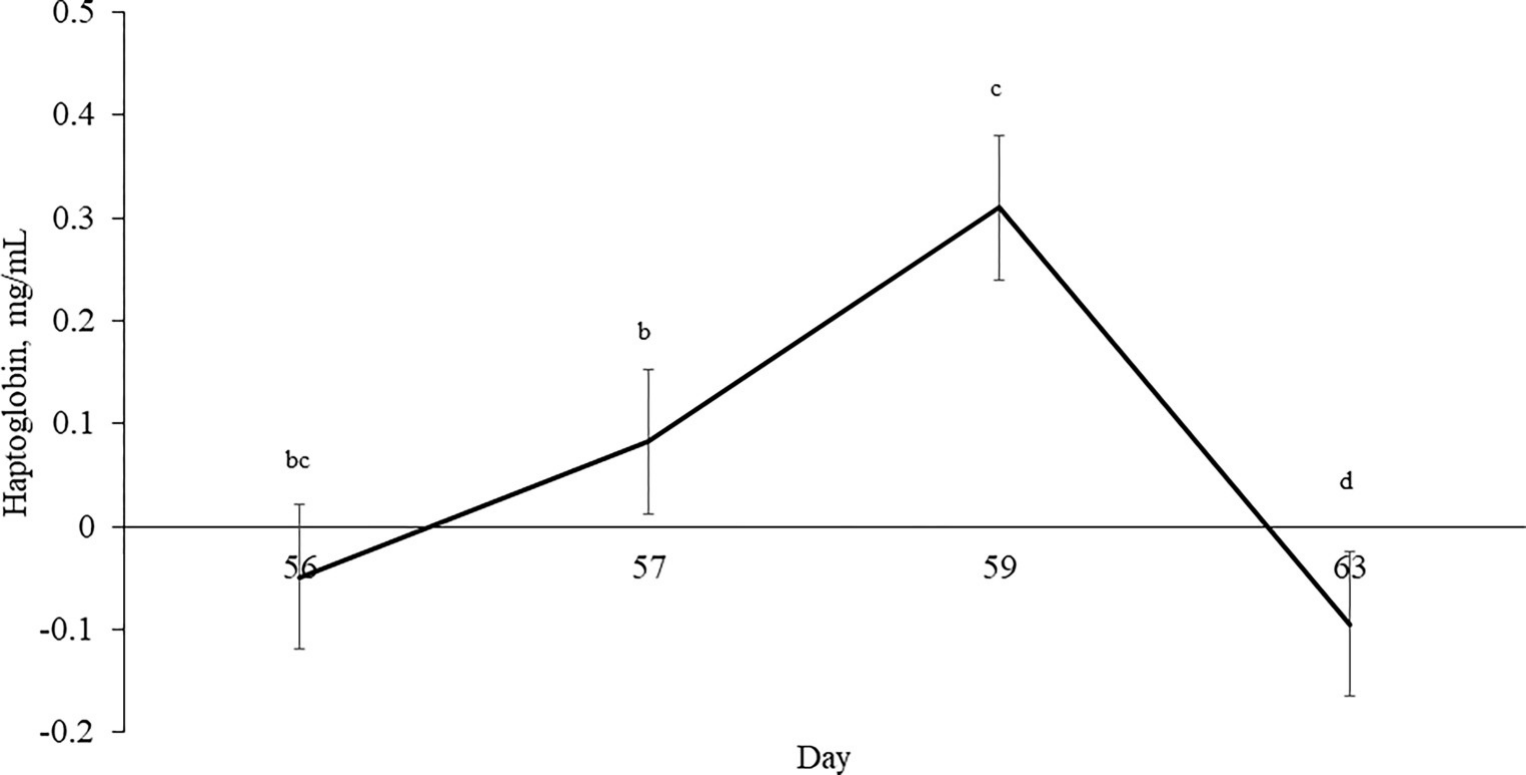
Effect of day (*P* < 0.001; SEM = 2.458) on plasma haptoglobin concentrations during weaning of beef heifers. For 56-d prior to weaning, heifers grazed bahiagrass pastures with or without access to artificial shade (11× 7.3 m in length and 2.4 m high). Within day, means without a common superscript (a–d) differ (*P* ≤ 0.05).

Haptoglobin concentrations, used as an indicator of stress during weaning, were affected by day during the post-weaning phase (*P* < 0.001; Fig 5). Effects of BREED were detected for Hp (*P* = 0.02). However, means were negative values that are below the threshold for inflammation (-0.00847 vs. -0.125 for ANGUS and BRANGUS, respectively). No effects of SHADE were detected for Hp (*P* = 0.716).

### Subsequent calf performance

Gestation length was affected by the availability of shade (*P* = 0.02; Table 6). NO SHADE had 18 ± 4.5 d shorter gestation period compared to SHADE cows. However, no effects were observed in birth or weaning weights of subsequent offspring (*P* ≥ 0.25).

**Table 6:** Gestation length, birth weight, and weaning weight of calves born from ANGUS and BRANGUS cows that grazed bahiagrass pastures with or without access to artificial shade (11× 7.3 m in length and 2.4 m high) for 56-d (July to September) during early gestation.

	BREED			SHADE			*P*-value		
Item^1^	ANGUS	BRANGUS	SEM	SHADE	NO SHADE	SEM	BREED	SHADE	BREED × SHADE
Cows, *n*	23	22	-	23	22	-	-	-	-
Gestation length, d	282	283	4.5	292	274	4.5	0.83	0.02	0.42
Birth weight, kg	35	37	1.3	37	35	1.3	0.39	0.58	0.24
Weaning weight^1^, kg	270	264	8.1	272	262.5	8.1	0.65	0.43	0.25

^1^Forty-five cows calved 22 heifers and 23 bull calves.

## Discussion

Grazing beef cattle in tropical and subtropical regions are at risk to suffer from HS. Heat stress is observed when environmental conditions (e.g., high temperature and humidity) cause the total heat load of an animal to be greater than its capacity of heat dissipation [17]. When cattle are unable to effectively dissipate heat for the maintenance of homeothermy, body temperature rises [18]. The provision of shade is associated with reductions in body temperature in beef and dairy cattle [8, 19–22]. However, in the current study, Brangus and Angus cows with access to shade had similar vaginal temperatures compared to cows without access to shade. Our previous research showed that artificial shade provided during summer in Florida reduced vaginal temperature of grazing beef heifers in the first 3 wks of the experiment [8]. The maximum vaginal temperature difference between heifers with or without access to shade was 0.4°C and was achieved in the afternoon, whereas in the current experiment, the magnitude of differences was 0.3°C. Nevertheless, an increase in body temperature from 0800 to 1800 was observed regardless of access to shade. In the current experiment, THI was greater than the one reported in our previous research (9; 78 vs. 75, respectively), therefore, it is possible the shade provided was effective but not sufficient to reduce vaginal temperatures.

Prolactin is a homeorhetic hormone that is involved in the adaptive metabolic responses during HS [23]. Indeed, greater air temperature has been associated with increased circulating prolactin concentrations in cattle [24–28]; The exact mechanism that explains why prolactin concentrations increase during HS is still unknown [29]. Prolactin is linked to modifications in the intracellular response to HS by triggering heat shock protein gene expression [26]; it participates in mechanisms regulating the increased water and electrolyte demands that occur in heat-stressed animals [30]; and it mediates HS-induced hyperinsulinemia [29]. While the role of prolactin during HS is not well understood, prolactin is a potential indicator of heat tolerance in cattle [27]. Hence, herein we evaluated plasma concentrations of prolactin as an indicator of HS in beef cows. Regardless of access to shade, in the current study, increased solar radiation enhanced plasma concentrations of prolactin in lactating pregnant beef cows. Furthermore, Angus had greater concentrations of prolactin compared to Brangus cows. In general, *Bos indicus* or *Bos indicus-*crossbred cattle are more thermotolerant, which might explain the lower prolactin concentrations observed in our study. One of the first signs of HS is reduction in feed intake, which is possibly a strategy to minimize the heat increment of feeding [6]. However, despite lowered feed intake, heat-stressed cows have enhanced glucose clearance, and increased insulin concentrations coupled with lowered fat mobilization [31]. One of the hypotheses behind the mechanism of lowered fat mobilization and increased insulin concentration during HS is that β-oxidation of non-esterified fatty acids may produce more metabolic heat compared to carbohydrate oxidation [32]. Increased plasma concentrations of insulin are associated with activation and upregulation of heat shock proteins [33] and hyperprolactinemia [34]. Angus cows with access to shade had greater concentrations of insulin on d 14 of the study. When solar radiation was greater (d 14 to 28), shaded Angus cows had lowered insulin concentrations compared to cows without access to shade. It is possible that access to shade provided during the days with the highest solar radiation contributed to mitigate HS and consequently to reduce plasma concentrations of insulin. On the other hand, Brangus cows, regardless of access to shade and day of the study, maintained constant insulin concentrations compared to Angus. *Bos indicus-*crossbred cattle, such as Brangus, have better capacity to regulate body temperature due to enhanced capacity of heat loss in response to HS [35], hence, Brangus cows were able to maintain constant levels of insulin and prolactin compared to Angus cows. Changes in plasma concentrations of glucose depend on the intensity and duration of HS [34]. In the current study, glucose concentrations were greater for Angus than Brangus cows. Heat-stressed cow experiences postabsorptive metabolic changes that are characterized by increased insulin release, and an increase in glucose disposal [31]. The fact that Brangus cows had lower glucose concentrations compared to Angus cows can be explained by a constant insulin concentration throughout the study, resulting in a more efficient glucose utilization.

Forage intake was not measured in the current study; however, we were successful in maintaining similar HA within treatments. [36], reported that HA below 1.0 kg DM/kg of BW is often associated with lack of sufficient forage for ad libitum consumption. Our lowest HA was 1.6 kg DM/kg of BW throughout the study; therefore, not being the cause of weight loss, but instead, it could be cause by some degree of HS as demonstrated by increased insulin and prolactin concentrations in Angus cows. Probably, nutrients were diverted from preservation of BW toward maintaining euthermia, resulting in loss of BW and BCS. *Bos taurus* cattle are known for their impaired performance compared to *Bos indicus*-crossbred cattle when consuming low-quality roughages due to possibly difference in their ability to remove organic matter from the rumen [37]. Hence, in addition to the heat tolerance-capacity of Brangus cows compared to Angus, the digestion ability might also contribute to explaining the impaired performance of Angus when grazing low-quality bahiagrass pastures regardless of access to shade.

Nursing heifers with access to shade gained 0.14 kg/d more during the pre-weaning phase compared to heifers without access to shade. With an impact on BW of Brangus heifers, which were the lightest at weaning and at 14-d post-weaning. In our previous study [8] we demonstrated that heifers grazing warm-season forages with access to an artificial shade, gained 0.2 kg/d, whereas heifers without shade lost 0.02 kg/d. Enhanced growth performance for grazing beef heifers has been reported by Monn et al. [22], who observed an increase of 0.2 kg/d for beef heifers with access to shade compared to cows with no access to shade. Providing shade to grazing cattle may translate into improved growth performance because shade structures protect cattle from direct solar radiation exposure [38]. During the post-weaning phase, no additional differences were observed for ADG, and DMI, except for BW, resulting in a lack of compensatory growth of Brangus heifers even post-weaning.

Weaning, feedlot entry, and vaccination of beef cattle elicit an acute phase response that triggers hepatic acute phase proteins [39], including Hp. Therefore, we analyzed plasma concentrations of Hp as a marker of post-weaning inflammatory response of beef heifers. Plasma Hp concentrations peaked on d 3, and gradually decreased on d 7, relative to weaning, which agrees with previous studies [11, 40]. However, the lack of provision of artificial shade during the pre-weaning phase did not attenuate the inflammatory response of beef heifers during the weaning period.

Insulin-like growth factor I has anabolic functions under normal circumstances by stimulating growth [41]. The concentration of IGF-1 has been found to decrease under undernourishment [42] and by HS exposure [43]. Angus heifers without access to shade had a reduction in IGF-1 concentrations of 63% at weaning, whereas heifers from Brangus cows had a reduction of 36% at the post-weaning phase. Lack of availability of shade reduced IGF-1 concentrations in heifers probably due to some degree of HS and/or reduction in feed intake. Insulin-like growth factor I impacts gonadotropin activity required for puberty achievement in beef heifers [44] and identifying strategies to enhance plasma IGF-1 during the pre-weaning phase may optimize future reproductive performance [45]. Limiting shade availability during the pre-weaning phase reduced IGF-1 plasma concentrations in growing heifers, which may cause long-lasting effects on growth and reproduction. Thus, more research is needed to understand the effects of HS during the pre-weaning phase and future outcomes on performance traits of beef heifers.

It is established that late-gestation cows exposed to HS have shorter gestation length [46] and give birth to lighter calves [47]. In the current study, gestation length, but not birth and weaning weights, was impacted by provision of shade, in which cows without access to shade during early gestation had shorter gestation length than cows with access to shade. Beef cows exposed to elevated temperatures during late gestation had shorter gestation length [46]. Increased body temperature of the cow results in increased temperature of the fetus and early initiation of parturition [46]. Exposure to HS in ewes during early to mid-gestation reduced birth weight of lambs by 20% compared with thermoneutral counterparts, but no impact on growth of lambs during their early postnatal life was observed [48]. Nevertheless, the newborn lambs presented altered pancreatic and adrenergic sensitivity. It is unclear why in the current study birth and weaning weights were not altered considering gestation length was 18-d shorter for cows without access shade. Pregnant cows exposed to HS have lower concentrations of circulating placental hormones, which cause impairment in the performance of the progeny [49]. However, it is still possible that other physiological mechanism, not evaluated in the current study, might have been altered, resulting in impaired performance of the offspring later in life.

In summary, providing artificial shade to pregnant-lactating beef cows positively impacted body weight gain of nursing heifers and Brangus cows while no positive effects were observed for Angus cows. The addition of artificial shade to grazing beef cow-calf pairs in tropical and subtropical climates can be an useful management strategy that can improve animal performance and profitability per unit of land area.
